# Impacts of the COVID-19 pandemic on the experiences of incarcerated pregnant people

**DOI:** 10.1186/s40352-024-00296-3

**Published:** 2024-10-19

**Authors:** L. Noël Marsh, Camille Kramer, Rebecca J. Shlafer, Carolyn B. Sufrin

**Affiliations:** 1 Department of Anthropology, University of Pittsburgh, Behavioral and Community Health Sciences, Wesley W. Posvar Hall, #3302, 230 S. Bouquet Street, Pittsburgh, PA 15213 USA; 2 Department of Gynecology and Obstetrics, Johns Hopkins School of Medicine, Center for Medical Humanities and Social Medicine, 4940 Eastern Ave, Baltimore, MD 21224 USA; 3 Department of Pediatrics, University of Minnesota, 717 Delaware Street SE, Room 382, Minneapolis, MN 55414 USA

**Keywords:** COVID-19, Incarceration, Pregnancy, Parenting, Quarantine

## Abstract

**Background:**

The COVID-19 pandemic disproportionately impacted incarcerated populations, yet few studies have investigated the specific effects on incarcerated pregnant people. This study compares pregnant people’s experiences of pregnancy and parenting in prison before and during the pandemic in order to explore the impacts of COVID-19 on this population.

**Methods:**

We conducted semi-structured interviews with pregnant people at a state prison as part of a larger study on pregnant people’s experiences during incarceration. Interviews explored participants’ experiences and decision-making related to pregnancy and parenting while incarcerated. This secondary analysis compared interviews conducted between June 2019 and March 2020 (pre-COVID-19) to interviews conducted between June and November 2020 (during COVID-19). Interviews conducted during the pandemic included questions about the impact of COVID-19 on participants’ experiences. Brief three and six-month follow-up interviews were conducted when possible.

**Results:**

COVID-19 introduced new stressors and exacerbated preexisting stressors around participants’ reproductive and parenting experiences. Three major themes emerged: 1) incarceration causes mental, emotional, and physical distress during pregnancy and parenting; 2) COVID-19 worsened conditions of incarceration, contributing to participants’ distress; and 3) the introduction of quarantine protocols during the pandemic felt uniquely punitive for pregnant and postpartum people.

**Conclusions:**

The COVID-19 pandemic was characterized as a major crisis and primary threat to public health, particularly for incarcerated individuals. Yet just as COVID-19 exacerbated preexisting disparities for marginalized, non-incarcerated communities, incarcerated pregnant people similarly described a “worsening” of already-intolerable conditions. The indiscriminate application of quarantine protocols for pregnant people reflects broader carceral logics of control that do not account for the wellbeing of pregnant and postpartum people and their infants, as evidenced by current practices of infant separation, a lack of support, and physically taxing living conditions.

## Background

In April 2020, the death of Andrea Circle Bear drew attention to the heightened mortality and morbidity risks for pregnant people who contracted COVID-19 while incarcerated (Bogel-Burroughs & Swales, 2020). At that point in the pandemic, United States (U.S.) prisons and jails had emerged as major hotspots for COVID-19 infections (Williams et al., 2020). By the summer of 2020 some evidence suggested that pregnant people were at higher risk of morbidity and mortality from COVID-19, especially when compounded by underlying chronic conditions (Mandavilli, 2020). The Centers for Disease Control and Prevention (CDC) later confirmed this with additional evidence (Zambrano et al., 2020).

Pregnant and postpartum people make up a relatively small percentage of incarcerated people in the U.S. Yet, prior to the pandemic, an estimated 58,000 pregnant people were admitted to prisons and jails each year (Sufrin et al., 2019, 2020). Media and scholars devoted some attention to the impacts of COVID-19 on birthing people’s mental and physical health in community settings (Jin & Murray, 2023; Tomfohr-Madsen et al., 2021). At the same time, scholars, policymakers, and advocates were increasingly concerned by the rising rates of COVID-19 infections and deaths in carceral facilities, especially given the higher burden of infectious disease among incarcerated populations (Barnert et al., 2021; Dumont et al., 2012; Saloner et al., 2020). Rapid COVID-19 transmission and worse health outcomes for incarcerated people prompted many scholars and advocates to call for decarceration as a pandemic control strategy, but most of these calls did not mention pregnant people (Altibi et al., 2021; NASEM, 2020; Ransom & Feuer, 2020). While the American College of Obstetrics and Gynecology (ACOG) recommended diverting pregnant people from custody, among other risk-reduction measures, it is unclear whether incarcerated pregnant people’s needs were adequately recognized and met by carceral institutions during the pandemic (ACOG, 2021b; Hutchinson-Colas & Sachdev, 2021; Kramer et al., 2022). Additionally, decarceration was not widely or effectively implemented, and preliminary data suggests that the number of incarcerated people is, as of 2021, rising again, especially in jails (Carson & Kluckow, 2023; NASEM, 2020). It is thus likely that increasing numbers of pregnant people are once again experiencing incarceration, though this cannot be confirmed since carceral facilities do not systematically collect or publish data about residents’ pregnancy status.

From a reproductive justice perspective, mass incarceration in the U.S. and its intersections with white supremacy, structural racism, institutionalized sexism, and economic exploitation help drive conditions of reproductive oppression against marginalized populations (Hayes et al., 2020; Ross et al., 2017). Reproductive justice describes the human rights to not have children, to have children and birth in safe conditions, and to parent in dignity and safety. Independently of COVID-19, incarceration inherently disrupts people’s rights to all of these, due in part to substandard reproductive health care and direct limitations on their abilities to be with their children (Hayes et al., 2020; Ross et al., 2017). Not only are Black and Brown people disproportionately incarcerated and suffer worse perinatal morbidity and mortality than white individuals, but they also have been disproportionately impacted by COVID-19 (Carson, 2022; CDC, 2023; Gibson, 2020; Romano et al., 2021).

Most carceral facilities do not adequately address women’s, transgender, and/or gender-nonconforming individuals’ unique reproductive health needs (ACOG, 2021a; Kelsey et al., 2017; Kramer et al., 2023). Incarcerated pregnant people are at increased risk of adverse health outcomes due to multiple factors, both preexisting and inherent to carceral contexts (ACOG, 2021a). Meta-syntheses of incarcerated pregnant people’s experiences pre-COVID-19 document psychologically distressing conditions, limited support and programming, and variable access to pregnancy care that threaten their overall physical and mental wellbeing (Cavanagh et al., 2022; Ferszt & Clarke, 2012; Kelsey et al., 2017; Tsuda‐McCaie & Kotera, 2022). While some facilities made efforts to enhance telehealth services and waive medical visit copays during the pandemic, COVID-related disruptions to operations and care likely disproportionately impacted pregnant people, especially since their health needs are time-sensitive and frequent (Kramer et al., 2022). Additionally, some facilities’ use of disciplinary spaces (e.g., solitary confinement cells) for quarantine poses specific health hazards for pregnant and postpartum people (Kramer et al., 2022). In a study of non-pregnant incarcerated people, such practices detrimentally conflated public health practices with punishment (Song et al., 2023); however, no studies to date have documented the lived experiences of pregnant and postpartum people in custody to examinethe impacts of quarantine during COVID-19 on this unique population during COVID-19.

The invisibility of pregnant and postpartum people in U.S. prisons and jails negatively impacts not only their health and wellbeing, but also that of their children and families (Hayes et al., 2020; Shlafer et al., 2019). Their omission from public discourse and efforts during a global health crisis, especially when considering intersecting racial/ethnic disparities, is deeply concerning (Hutchinson-Colas & Sachdev, 2021). While there are some qualitative studies of incarcerated people’s experiences of being in custody during COVID-19 (Pettus-Davis et al., 2021; Song et al., 2023), data are lacking about pregnant people’s experiences. This paper aims to start filling that gap by comparing incarcerated pregnant people’s experiences of pregnancy and parenting before and during COVID-19.

## Methods

### Overall study design

This analysis was a part of a larger study that focused on the experiences and pregnancy-related decision-making around abortion, birth, and infant placement of incarcerated pregnant people at two state prisons and two jails in two states (Sufrin et al., 2023). The present analysis focuses on the one study site that allowed us to resume interviews after the pandemic hit in March 2020. We conducted semi-structured, qualitative interviews with pregnant individuals at this study prison from June 2019 to November 2020. Because the study was ongoing as the COVID-19 pandemic emerged, we expanded its aims to include how COVID-19 impacted experiences of being incarcerated and pregnant.

We had a preexisting research relationship with the study prison, and they agreed to be a site for the larger study on pregnant people’s experiences and decision-making. We conducted 6 interviews between June 2019 and early March 2020 before the COVID-19 shutdowns began in late March 2020. After a brief pause (3 months) due to the shutdown, the prison allowed us to shift from in-person study activities to virtual recruitment and interviews. We conducted an additional 6 interviews virtually between June and November 2020, for a total of *N* = 12 participants at this site.

### Recruitment and study procedures

The prison identified a study contact in the medical unit to serve as the liaison between our team and the facility. The study liaison notified our team when a pregnant person who was interested in learning about the study arrived at the facility. A research team member then met with the person to assess eligibility and explain the study. If they were eligible and interested in participation, we conducted the interview at least 3 days later in a private space at the facility. The lag time helped ensure that participants had adequate time to consider their participation and avoid undue pressure. For in-person interviews, facility staff escorted the research team member to a private room upon arrival at the facility. The research team member only initiated the interview once alone in the room with the participant and after confirming that no one could overhear the participant’s responses (e.g., closing the door/windows). Facility staff were not privy to the specific questions asked of participants in the interview guide.

For remote interviews, facility staff escorted participants to a private space (usually an unused staff office), logged them into Zoom, left the room, and closed the door. The research team member explicitly asked remote participants whether they were alone and felt comfortable with privacy levels before proceeding with the interview.

Eligibility criteria included currently pregnant, English-speaking individuals aged 18 years or older. Participants were compensated for their interview in custody with a pregnancy resource book, per prison guidelines of allowable compensation. We stopped recruiting participants after we reached thematic saturation for the parent study. Interviews were audio recorded and transcribed verbatim via a third-party transcription service, and all identifying information removed. Finally, we collected contact information and attempted to reach participants 3 and 6 months after their due dates for a brief, structured follow-up interview to learn about their pregnancy outcomes. We were unable to follow up with participants who did not respond after three contact attempts, or whose contact information was no longer valid. If a participant was back in the community during their follow-up calls, they were compensated with a $15 gift card to a general merchandise retailer for each call. Three pre-COVID-19 participants took part in a 6-month follow-up interview; we were also able to contact three COVID-19 participants for a 3-month and 6-month follow-up interview each.

### Interview guide

We grounded the interview guide in conceptual frameworks and theories of reproductive justice and the ways in which individuals’ decision-making and care access are shaped by the coercive dynamics, punitive environments, and limited autonomy inherent to U.S. incarceration (Hayes et al., 2020; Ross et al., 2017). Questions were also informed by a prior ethnographic study of incarcerated pregnant people and a study of abortion decisions among people obtaining prenatal care (Roberts et al., 2019; Sufrin, 2017). The interview guide addressed multiple facets of incarcerated pregnant people’s experiences and decision-making, including access to medical care and services, housing, social support, pregnancy continuity or termination, and infant placement. Follow up interview questions addressed incarceration, pregnancy outcomes, and future plans. We obtained feedback on the original interview guide from community members who had experienced pregnancy during a period of incarceration.

After March 2020, we adapted the interview guide to include additional questions about COVID-19’s impact on pregnant people’s experiences in custody with perinatal care and services, plans for their pregnancy, pregnancy behaviors and childbirth, housing, and in an open-ended fashion, any other experiences participants wanted to share (see Table 1). These additional COVID-19 related questions were informed by emerging evidence of the impact of the pandemic on general prison medical care and housing protocols. These questions were also theoretically grounded in the coercive and controlling aspects of incarceration, and that these might intersect with prison responses to a new public health threat. While we did not specifically ask about COVID-19 in the follow-up interviews, the last open-ended question (e.g., Is there anything you want to add?) allowed participants to describe birth and postpartum experiences, as well as COVID-19-related impacts.

**Table 1:** Questions added to the interview guide to address COVID-19

Topic	Question(s) added
Pregnancy medical care	This study is not focused on COVID but we want to acknowledge that COVID may have greatly impacted your time and care in custody and how you think about your pregnancy. How has COVID impacted pre-natal care and other services available to you while in custody (either in jail or here at the prison)? Probe: Was any service not available to you because of COVID?
Pregnancy decisions	How has COVID-19 impacted your plans for your pregnancy?
	Has anyone talked to you specifically about COVID regarding your pregnancy? [Probe: tell me more, do you feel like you received adequate information about COVID]
	[If participant discloses asking about obtaining an abortion] Do you think that COVID has affected the ability to get an abortion?
	How has COVID affected your thoughts about the child birthing process and/or your behaviors for the rest of your pregnancy? Have you gotten any information about what to expect at the hospital while in labor or after you give birth?
	Has anything changed about your housing because of COVID? If yes, how so?
Final questions	Is there anything else you’d like to share about what it’s like being incarcerated while pregnant during COVID? If in custody before COVID, has anything changed?

### Data analysis

When first conceptualizing this paper to answer the question, “How did COVID-19 impact the experiences of incarcerated, pregnant individuals?” we planned to focus only on the six participants we interviewed during COVID-19. However, after reviewing the COVID-19 transcripts, we determined that incorporating the six pre-COVID-19 interviews and follow-up summaries would add valuable comparative data and context for understanding. We then revised the guiding research question to be, “In order to understand the impact of the pandemic on incarcerated pregnant people, how do the experiences of incarcerated pregnant people compare pre- vs. during COVID-19?”.

We approached data analysis from a critical realist framework, which incorporates social context and the subjectivity of individual experience into a multifaceted analysis of empirical reality (Fletcher, 2017). We utilized both inductive and deductive approaches to thematically analyze all the available transcripts and follow-up interview data from this study site (Braun & Clarke, 2006). All authors reviewed interview transcripts. The first author coded all interview transcripts (*N* = 12) and follow-up interview summaries (*N* = 9) in NVivo13 (QSR International, 2020) using pre-specified domains corresponding to the interview guide. She then used a combination of descriptive and concept coding on the COVID-19 transcripts, then the pre-pandemic transcripts, to create a codebook and assess whether pandemic participants described notable differences in their experiences compared to pre-pandemic participants, even when not explicitly discussing COVID-19 (Saldaña, 2016). Memos were iteratively written in tandem with coding to reflect on codes and patterns within them and reflect on researcher positionality during analysis. The first author then refined the codes to focus on categories of experience (e.g., housing, quarantine, parenting and reproduction) that COVID-19 impacted and visualized emergent themes in several iterations of a concept map. The study team met regularly during analysis and writing to discuss findings and emergent themes.

### Setting

The study site is a multi-security-level state women’s prison with an average population of approximately 2,300 individuals in 2022. When individuals entered the study prison before the pandemic, they were initially housed in “Admissions” for 30–60 days where they went through intake and classificatory processes, including medical screenings. They were then transferred into the prison’s General Population; certain populations (such as pregnant people) might be assigned to special housing. According to state Department of Corrections (DOC) reports publicly available on their website, an average of 27 pregnant people were in DOC custody from June 2019 – February 2020, and an average of 18 pregnant people were in DOC custody from March – November 2020. Pregnant people at this prison can apply for the “nursery” program, which allows them to reside postpartum in a special housing unit with their newborn. Pregnant people who do not qualify for the nursery program are separated from their infants shortly after birth. At the beginning of the COVID-19 pandemic the prison suspended in-person programming and visitation, though the nursery program continued to operate. This state also passed an “anti-shackling” law during the course of our research study, banning the use of restraints throughout pregnancy, birth, and up to 6 weeks postpartum.

### Ethical considerations

The Johns Hopkins School of Medicine Institutional Review Board approved this study, and we followed the research approval processes of the prison. Our recruitment protocol took special care to avoid coerced participation. We conducted interviews in a private space away from facility staff. All names in the results are pseudonyms. The prison did not allow us to provide direct compensation to participants in custody, but we did provide them with a resource book about pregnancy-related health and wellness designed specifically for incarcerated people.

## Results

All 12 participants identified as women.^1^ Most (83%) participants identified as white. They ranged in age from 21 to 35 years old (*M* age = 27.8) and at the time of the interview, ranged in gestational age from first to third trimesters. All had been housed in the state prison for 2 months or less at the time of the interview (see Table 2). At the time of the interview, no participants reported having contracted COVID-19 during their time in prison. Out of the six individuals who participated in follow-up interviews, all but one was still in custody at the time of the interview (Table 2).

**Table 2:** Participant demographics and other characteristics

**Characteristic**	**n (%)**
Participated in a 3-month follow-up interview	3 (25)
Participated in a 6-month follow-up interview	6 (50)
Average age in years (minimum, maximum)	27.8 (21, 35)
Median gestational age at the time of interview in weeks (minimum, maximum)	20.6 (11, 39)^a^
Median duration of current incarceration at study site in days at the time of interview (minimum, maximum)	20.5 (13, 60)
Previously incarcerated	10 (83)
Housing location in the prison at first interview	
Admissions	9 (75)
General population	3 (25)
Race (number in each category)	
Black, non-Hispanic	1 (9)
White, non-Hispanic	10 (83)
Native American, Hispanic	1 (9)
Highest education level	
Some high school	3 (25)
High school diploma/graduate equivalency degree (GED)	6 (50)
Some college	3 (25)
Housing status prior to incarceration	
Stable housing (lived with family, on their own, with partner)	12 (100)
No stable housing	0 (0)
Employment/source of income	
Employed	4 (33)
Unemployed	7 (58)
Drug trade	1 (9)
Number of women who have given birth to at least one child	10 (83)
Average number of children they have given birth to (minimum, maximum)	2 (1, 5)
Number who had been pregnant in jail/prison during a prior pregnancy	2 (17)
Gave birth in custody with prior pregnancy	1 (9)
Incarceration status at birth^b^	*N* = 6
Prison/jail	5 (83)
Community (post-release)	1 (17)

^a^Data for one participant was missing and not included in the calculation

^b^We were able to contact six participants after they had given birth

Overall, interviews revealed that participants’ reproductive and parenting experiences were extremely stressful due to their incarceration, and COVID-19 both exacerbated preexisting stressors and introduced new ones. Three major themes emerged: 1) incarceration causes mental, emotional, and physical distress during pregnancy and parenting; 2) COVID-19 worsened conditions of incarceration, contributing to participants’ distress; and 3) the introduction of quarantine protocols during the pandemic felt uniquely punitive for pregnant and postpartum people. Representative quotes appear in the sections below and in Table 3.

**Table 3:** Exemplary quotes

Theme	
**Subtheme**	**Representative quote(s)**
**(1) Incarceration causes mental, emotional, and physical distress during pregnancy and parenting**	
* Feelings of anxiety and powerlessness*	1. “I felt like it didn’t – the doctor didn’t really want to hear what I had to say. I got Raynaud’s disease, so I’m supposed to have medicine. I don’t have no more medicine and they don’t even want to hear that. Like I don’t know what I’m talking about. I have a disease, so I think I know what I’m talking about. They just didn’t want to hear what I had to say. It’s really irritating. […] She just was telling me what she knows, and she don’t know what she’s talking about, I don’t think, at all. You can’t tell someone that without being rude, so I don’t know what I’m going to do about that situation. […] I don’t know what to do when you’re just completely shot down by staff here.” – Jessica, during COVID-19
* A lack of information amplified participants’ fears*	2. “They haven’t, like, went into depth or told me, like, ‘So, when you go into labor here this is what we’re going to do. You’re going to go through this process, or you’re going to be squaded out.’ Or, you know, ‘This is what’s going to happen.’ They haven’t really went into depth about those things. I don’t know; I’m scared to go into labor. I really am.”—Kathy, pre-COVID-19 3. “I have no idea [what to expect for pregnancy care]. I just know that I get my prenatal vitamins, I get my seizure meds. I do know that before going to deliver, I will be put in the hole to be quarantined, which isn’t ideal, at all. It’s pretty scary, actually.”—Aylen, during COVID-19
* Fears of losing child custody due to incarceration*	4. “They kind of threatened [me and my partner], that Children’s Services would get involved. I’m thinking that can’t legally be right. Because I’m in here, that doesn’t mean that gives you the right that I have a Children’s Services case.”—Maya, pre-COVID-19
* Infant separation triggers tremendous anxiety*	5. “I mean, it’s very—it’s stressful. It’s really stressful not knowing what’s happening right now. I can’t imagine not having her—having her and having to give her up as soon as I have her. Whether it be to my family or not, that would be absolutely horrible. I think that a newborn really needs a mother’s bond. Especially right when she’s born. So, it would just be very rough if I have to just give her up as soon as I have her. It’s very stressful until I know what’s going on for sure.”—Eva, during COVID-19
* Worries about mental health impacts of infant separation*	6. “You’ll come back here right after you give birth – two days after – and you’ll be back out into [general] population and just continue on with your prison sentence, like nothing’s happening. So, I’m a little uneasy about that, if I do end up having to give birth here and come back, of how I’m going to adjust with that, and how they’re going to deal with my post-partum, if I got through that, and stuff like that. They haven’t really clarified how they’re going to do that. Will I be in counseling? Will I—you know? What do you guys have to offer after the baby, you know? Do I get extra, you know, mental care with that? “—Kathy, pre-COVID-19
* Separation is counterproductive to rehabilitation*	7. “I think it’s ridiculous. Because I feel like—I mean, we’re here by ourselves, and, like, it’s going to cause severe post-partum [depression]. Like, only being—because, after we have birth, we’re only allowed 24 h with the baby until it’s taken. So, I feel like this place, like, it’s trying—they send you here to make yourself a better person. To find you or to make yourself better, and by taking away something that you care about—it’s not right. [Cries] And I feel like everybody should get a chance.”—Hannah, pre-COVID-19 8. “Considering it is my first baby, you know, I think it would do more harm than help me if you separated me. Yeah, because you only get to spend 48 h with your baby before it gets taken. So I don’t think that would, you know, help me in my long-term life of just messing up.”—Kathy, pre-COVID-19
**(2) COVID-19 worsened conditions of incarceration, contributing to participants’ distress**	
* Lack of information about COVID-19 and pregnancy*	9. “[Any information about COVID-19 and pregnancy specifically is] word of mouth. So, I mean, they might say a few things here or there. But it’s passed along the lines, so you kind of just, you know, got to decipher what – what is more just rumored and what’s, you know, real.”—Gina, during COVID-19
* COVID-19 amplifies isolation and a lack of support*	10. “Honestly, I feel like the COVID is making everything a lot harder. I feel like we don’t have nearly the amount of support that we would have. I have been told by several women that, usually, when COVID’s not here, we have support groups. There’s classes. Visitors can come in. If my mom and my kids could come and see me and we could actually sit down and talk about things, rather than over the 5 min or 15-min phone calls, when I can get to a phone, that makes it really hard. If I could just have time with her to sit down and actually have a full discussion about the way that I’m feeling, it would’ve made this whole process a lot easier. The mail right now is even messed up because of the COVID. Where it’s usually a week behind, it’s a month behind, so conversating between me and her father – we’ve only had a short amount of time to get stuff. I don’t want to make any decisions without him being involved. Decisions where I’ve had to just go ahead and make, which I’m not used to.” Aylen, during COVID-19
* COVID worsens living conditions*	11. “The COVID’s making it a lot worse. A lot worse. We don’t get the yard time and stuff like that that we typically would. We’re literally locked in the building. The doors can’t be opened right now. Our doors aren’t opened, so even with fans on it’s not cooling anything off. … These women are used to going to classes every day and they’re not able to do that, now. So, they’re frustrated. They’re hot. We’ve got older people, people with breathing problems and stuff like that that are on oxygen and stuff that are uncomfortable and hot and just – you combine that and put that around each other for long enough with no outlet and then it becomes a hostile environment…. [W]hen you don’t have the outlets and you don’t have the programming and stuff like that, you get enough women in a building and that gets that stressful. They start not to feel like they have nothing to lose, which makes it a scarier environment.”—Aylen, during COVID-19
**(3) The introduction of quarantine protocols during the pandemic felt uniquely punitive for pregnant and postpartum people**	
* Quarantine exacerbates physical and emotional distress*	12. “I know when we was in quarantine it was absolutely horrible. The stress was beyond unimaginable. [Other people] don’t care [that you’re pregnant]. They are always arguing. Always. They’ll argue with whoever they want. They don’t care. It’s just stressful. You got to listen to them arguing 24/7 from the time you get up to the time you go to sleep… Everybody’s so close together. There’s no air conditioning. … I was sweating nonstop. I was lightheaded because I was sweating so much, and I was so hot. I’m still pregnant. It was absolute horrible.”—Eva, during COVID-19
* Quarantine feels like punishment*	13. “[Quarantine after birth feels] almost as if [we’re] being punished, which I don’t believe is fair. Especially after having the baby, after giving birth to, you know, and having to hand your baby over. And then, having to come back, and spend in isolation for 14 days by yourself…. I feel like it’s probably not the smartest decision.”—Gina, during COVID-19 14. “I do know that before going to deliver, I will be put in the hole to be quarantined, which isn’t ideal, at all….That’s where they put people that have tested positive for the COVID. That’s also where they put people that they can’t control, obviously.”—Aylen, during COVID-19

### Incarceration causes mental, emotional, and physical distress during pregnancy and parenting

Participants both before and during COVID-19 expressed intense distress about how incarceration would affect various aspects of pregnancy, birth, and parenting. Feelings of anxiety, uncertainty, fear, and powerlessness pervaded their interviews, such as when Maya (pre-COVID-19) said, “There’s nothing you can do, you have no power here.” While some participants did report small moments of positivity – receiving social support from other pregnant women in the prison or supportive family members, and a few positive interactions with prison staff – overall, participants were “scared” and uncertain about what to expect, since “this is prison.” Two participants even described briefly considering abortion due to the heightened stress of being pregnant and incarcerated; however, the one who was incarcerated during COVID-19 did not mention the pandemic as a factor in her decision-making. Several distrusted or had negative experiences with the quality of the medical care in the facility. Difficulties receiving medication, feeling like medical providers dismissed their concerns, or not receiving information about their test results or care plans contributed to participants’ sense of powerlessness, both before and during COVID-19 (Table 3, Quote 1).

Bridget (pre-COVID-19) stated, “I feel like sometimes you can’t get answers,” even when asking pointed questions about her medical care. When the interviewer asked them whether they had considered genetic screening in pregnancy, for instance, Kathy (pre-COVID-19) and Beth (during COVID-19) had no idea that that was an option for them. This lack of information about what to expect, especially around giving birth, amplified participants’ fears (Table 3, Quote 2–3). Several women worried about the physical experience of going into labor, as well as the possibility of going through labor and birth “alone” and/or in shackles. Participants reported mixed expectations of whether a support companion (such as a parent or partner) would be permitted to attend their birth. As Eva (during COVID-19) stated, “I don’t know if I’m allowed to have anybody there [during labor] or what, but I’m just scared to death.” In order to alleviate her fears, Kathy described asking “other girls” who had been pregnant before “what they recommend,” and “that’s basically where I’m getting a lot of information about my pregnancy.”

Additionally, all participants perceived their living conditions as significant sources of stress, and several worried about the impacts the environment would have on their own health and that of their fetus. Participants from both cohorts living in Admissions described the housing unit as an extremely stressful, chaotic environment (“kind of a madhouse”) with no privacy and limited accommodations for pregnant people. For example, Bridget (pre-COVID-19) stated that during fights, “[The CO’s] come in, they pepper spray, there’s no precautions for the pregnant women. It’s not, ‘Hey, we get those out before any of that.’ […].” In both Admissions and the “honors” dorm, where pregnant people were housed in the main prison, participants also described intense heat during the summer months with no air conditioning, limited access to ice and cold water, no fans, and even water getting too hot to shower in.

A significant source of anxiety for all participants was the placement of their newborns, especially if they still hoped to be accepted into the nursery program, Others feared losing custody of their child as an eventual outcome of their immediate separation (Table 3, Quote 4). Although the prison in this study did have a nursery program, most participants both before and during COVID-19 did not expect they would meet eligibility criteria to be admitted to it, and thus anticipated some length of separation from their newborns.^2^ Every single participant described how separation from their infants, if they were not released before giving birth or accepted into the nursery program, would affect them negatively, along with the stress of having to find someone in the community (family member or friend) to care for their baby. Although several participants were currently separated from other older children, the idea of being separated almost immediately after birth felt acutely painful (Table 3, Quote 5). In her six-month follow-up interview, Maya (pre-COVID-19) described feeling “traumatized” by the separation from her infant, adding, “I don’t ever want to experience that ripped apart feeling again.”

The mental health impacts of being separated from their newborns weighed on participants in both cohorts (Table 3, Quote 6). When reflecting on the impending separation from their infants, both Kathy and Hannah (pre-COVID-19) brought up the supposedly rehabilitative mission of “correctional” facilities, and how infant separation counteracts any rehabilitative potential (Table 3, Quotes 7–8). As Hannah stated, “the punishment is, like, worse in the long run. Because, like, at the end of the day, like, you’re taking away my kid.” This separation-as-punishment felt, to Hannah, hugely disproportionate to her crime, which was not related to children. Additionally, participants felt that there was no support for postpartum women if they were not accepted into the nursery program. In their follow-up interviews, both Bridget (pre-COVID-19) and Hailey (during COVID-19) expressed the desire that the prison “keep the moms inside [housed] together for support” instead of separating them.

### COVID-19 worsened conditions of incarceration, contributing to participants’ distress

COVID-19 participants reported additional stresses due to the pandemic and the prison’s responses to it. Fears of exposure with potential harm to their babies and a lack of information about COVID-19 specifically exacerbated pandemic participants’ anxieties about the synergistic distress of being incarcerated, pregnant, and birthing during a pandemic. Hailey, who was staying in Admissions where people kept testing positive for COVID-19, feared contracting the disease and how it might impact her baby: “Well, it scares me. If I test positive, if she’s going to get it and how it’s going to affect her. If she’s born, like—I don’t know how that works, you know? It worries me.” While pre-pandemic participants also worried about giving birth “alone,” during COVID-19 it was unclear whether hospital^3^ or prison policies forbade support persons from attending the birth. The pandemic may also have impacted the amount of time participants spent with their newborns in the hospital, as Aylen reported, but this was unclear since Bridget (pre-COVID-19) also reported getting “no physical bonding time” with her baby in the hospital due to a flu diagnosis.

In addition to heightened fears about negative pregnancy care and outcomes, pandemic participants bemoaned a lack of information about how COVID-19 affects pregnant people specifically (Table 3, Quote 9). Jessica, for example, was told by correctional officers that the correctional officers’ [CO] ages (50 s and 60 s) put them at “higher risk” for negative outcomes, but no one informed her of any risks from COVID-19 to her pregnancy. Instead, she learned from a news segment about a pregnant woman transferring COVID-19 to her baby. Though a lack of information about medical care was not new, participants felt that it was much harder to get information because of the pandemic and how staff attention was diverted as a result. Jessica had seen a doctor for a general check-up in the prison (as opposed to the county jail, where “you really can’t get anything because it’s ‘COVID, COVID, COVID’”), but she still said, “They’re focusing on the COVID and not our health, I feel like. We aren’t getting any information on – well, I’m sure we will, but any information about what’s going on with our pregnancies and what our options are and things like that – because they’re worried about quarantining us and things like that.” Jessica perceived that the prison staff’s focus on COVID-19 meant that they were neglecting her health and pregnancy.

Pandemic restrictions such as facility-wide lockdowns (being confined to housing unit for days at a time and the inability to go outside), the cessation of programming and visitation, the introduction of quarantine protocols, and other disruptions (such as to mail service) also exacerbated participants’ mental and physical distress (Table 3, Quote 10). Participants felt like they had less social and emotional support, while others reported increased isolation. Mental and physical conditions compounded each other’s effects; while excessive heat, for example, was already an issue prior to the pandemic, Aylen worried that the combination of heat, lockdowns, stress, and a lack of programming would deleteriously affect residents’ mental and emotional health since “we’re literally locked in the building” (Table 3, Quote 11).

Pandemic restrictions had other unforeseen consequences as well, such as on Beth, who needed to complete a specific program before leaving the prison. Since the program was unavailable due to COVID-19 lockdowns, she would not be released prior to giving birth. Beth did not have a family member or partner who could care for her infant, and reported being told, “if I couldn’t find anyone to take my child that I would have to give [it] up for adoption,” which she did not want to do.

The pandemic also exacerbated both subtle and overt forms of emotional separation. The cessation of all in-person visitation prevented mothers from seeing or holding their infants after returning to prison. In her 3-month follow-up interview, Hailey, during COVID-19, noted that it would have been easier to cope with the separation if her infant and caregiver could have visited, as phone calls are ineffective for connecting with preverbal babies. At her six-month follow-up interview, Hailey still had not seen her baby in person, and reported that pictures “just aren’t the same.”

### The introduction of quarantine protocols during the pandemic felt uniquely punitive for pregnant and postpartum people

Although it was only one of several measures introduced to restrict the spread of COVID-19, quarantine practices had significantly deleterious effects on these pregnant participants. They reported undergoing quarantine when entering and exiting the prison, including when they returned to the facility after each off-site prenatal appointment or childbirth hospitalization. The length and location of quarantine changed over time, especially as COVID-19 tests became more readily available, and participants recognized that quarantine protocols were in place to mitigate the spread and impact of COVID-19. However, quarantine conditions still felt distinctly punitive to pregnant and postpartum people since they repeatedly had to leave the facility for medical care due to their pregnancy (and eventually postpartum) status.

While pandemic participants described different types of quarantine depending on their housing placement (Admissions vs. General Population housing) at the time of their interview, they all reported confusion and uncertainty over quarantine protocols. Sometimes participants were told conflicting information, while, to others, protocols seemed to change over time without explanation. In June 2020, for example, Gina was told that she would be placed in solitary confinement for quarantine after returning from the hospital to the prison post-birth, while in August 2020 Jessica was told “that if we were ever sent to [the hospital] – that we have to be quarantined for three days in C Corridor, which is another building, here.” Jessica stated, “I think it’s a big mess. They don’t really know what they’re doing, so we’re like their guinea pigs. That’s how it feels.”

Both group and individual modes of quarantine felt physically and mentally intolerable for participants. Eva and Jessica, for example, found the heat, overcrowding, and social stress of group quarantine distinctly harder because of their pregnancies (Table 3, Quote 12). Hailey, whose stay in Admissions quarantine was extended each time someone else in the group tested positive, was frustrated that she could not access the routine services and accommodations that people in General Population had access to (including more freedom of movement, the ability to order commissary, more food for pregnant individuals, and more privacy). She wanted to get out of Admissions because she could not buy food from the commissary and was “starving,” as the limited food at scheduled prison mealtimes were inadequate for her pregnancy-related hunger.

Gina described how individual quarantine practices also lacked basic consideration for pregnant people’s specific needs: “the way they’re handling things, especially if you’re taken out of here for any reason, to go to – for certain tests or after delivering your baby, once you return they are quarantining you for 14 days, just to be — as a precaution since you left the premises. But they’re putting girls in the hole, which is miserable. And you’re basically on lockdown.” Being “in the hole,” or in solitary confinement, was described as spending 23 h per day in a cell in the disciplinary building with limited or no access to communication devices (tablets or phones), or potentially any kind of medical or behavioral health support. Typically used for punishment, solitary confinement was regularly used for quarantine during COVID-19 according to participants (Table 3, Quotes 13–14).

Pandemic participants assumed that they would have to quarantine in isolation when returning to the prison after childbirth. They therefore understandably expressed concerns about how isolation would amplify their distress over being separated from their newborns. Aylen commented, “I’ve never been [in solitary confinement], so I don’t know what it’s like, but I know the stories that I hear are not good.” Some participants expressed fears of worsening mental illness (e.g., postpartum depression) after being separated from their newborn and placed in quarantine, alone, post-birth. Despite voicing her concerns to staff ahead of time, Aylen was unable to access mental health care during her post-birth quarantine. In her 3-month follow-up interview, Aylen reported that she had been quarantined in solitary confinement immediately postpartum, and for the first 12 days was only allowed one phone call per day. She was reportedly told by staff that she needed to “get [yourself] together” because “they weren’t gonna sit and listen to me cry.”

## Discussion

To our knowledge, this is the first study to describe the experiences of incarcerated pregnant people during the COVID-19 pandemic and compare to their pre-pandemic experiences. This is a population that has been largely elided in COVID-19 policy efforts. Notably, participants before and during the pandemic described very similar stressors regarding pregnancy and parenting in prison, and, for the latter, the virus itself was not necessarily their first concern. All participants described significant distress over separation from their newborns and other children, unsanitary and unpleasant living conditions that particularly affected them due to their pregnancies, coercive dynamics, a lack of information and autonomy, neglectful and/or iatrogenic medical care, and a lack of social support. None of our participants had contracted COVID-19 at the time of their interview, but all participants had only resided at the prison for two months or less, which could have influenced the degree to which COVID-19 was a concern during their interviews. Despite this limited time frame of experience, participants had experienced significant upheavals due to changes to facility operations meant to reduce the risk of disease transmission. Our findings therefore suggest that COVID-19 related stressors added to and exacerbated preexisting mental and physical stressors. Participants’ fears and sense of unfair punishment related to COVID-19 stemmed largely from their concerns about fetal/infant wellbeing, the frequency of quarantine due to their reproductive and health needs, and the compounding effects of infant separation and postpartum isolation.

Research in the U.S. and other countries has similarly found that incarcerated pregnant people seldom receive adequate medical care and social support, while also experiencing distress and/or (re)traumatization due to infant separation, unsuitable living conditions, and coercive dynamics (Breuer et al., 2021; Cavanagh et al., 2022; Sufrin et al., 2023). The United States is one of only four countries that routinely separates incarcerated birthing people and their newborn infants (Nair et al., 2021), which is extremely distressing for parents (Chambers, 2009). Regardless of the child’s age, parental incarceration negatively impacts children’s developmental and learning outcomes (Poehlmann-Tynan & Turney, 2021). In this study, the cessation of all in-person visitation and in-person programming exacerbated participants’ physical and emotional separation from their children; this was likely the case for all incarcerated pregnant people during the pandemic, as all state and federal prisons implemented similar restrictions (Dallaire et al., 2021; Muñiz et al., 2023).

As multiple studies with incarcerated, non-pregnant people have shown, the COVID-19 pandemic both exacerbated preexisting stressors and introduced new ones due fear of contracting the virus, facility-wide lockdowns, quarantine that mimicked solitary confinement, and a lack of information (Cassarino et al., 2023; Song et al., 2023). Many carceral facilities did attempt to increase residents’ access to digital communication methods (such as phone calls and email) during the pandemic, but our study suggests that this did not help postpartum people or those with nonverbal infants (Zielinski et al., 2022). This disconnect likely heightened pregnant and postpartum people’s isolation from their social networks, which is especially concerning in the immediate postpartum period when there is heightened risk of perinatal mood disorders. In the community, the pandemic greatly increased mental health issues and common perinatal mood disorders among pregnant and postpartum people, most of whom were not also dealing with infant separation (Jin & Murray, 2023; Moyer et al., 2020; Tomfohr-Madsen et al., 2021).

The experiences of pre-pandemic participants suggests that the prison already lacked adequate communication infrastructures between staff and residents, especially related to medical care, programming, and reproductive health. This pattern continued during the pandemic, with COVID-19 participants describing uncertainty and confusion about the disease itself, any risks it posed to pregnant people, and the facility’s disease management protocols. While we could not triangulate participants’ reports with facility data, Pettus-Davis et al. (2021) found that most of the 327 formerly incarcerated individuals they surveyed had gotten their information about COVID-19 through TV news or other programming, while only 1/3 of participants had received COVID-related information from prison staff. The fact that information and recommendations about COVID-19 changed quite rapidly, especially in the beginning of the pandemic, exacerbated challenges in communicating accurate information to the general public, leading to what some have called a concurrent “infodemic” (Scales et al., 2021). Unlike people in the community, however, incarcerated individuals in the U.S. have limited access to communication media (Reisdorf, 2023). Pregnant and postpartum people have additional information needs related to their health and that of their fetus, which, for our participants, compounded the stress of these informational lacunae.

Since participants described disorganized medical care both prior to and during the pandemic, it is not clear from the interviews whether COVID-19 negatively impacted participants’ abilities to access medical care. Yet participants’ reports indicate that the prison was generally ill-equipped to meet their needs, and even more so during the pandemic. This was especially evident in how pregnant and postpartum people were not given any special consideration with quarantine protocols. While intended to mitigate disease transmission, frequently quarantining pregnant people poses a double standard alongside the fact that carceral staff and administrators could enter and exit the facility on a daily basis without quarantining (Towers et al., 2022). Studies of other prisons during the pandemic also document the use of solitary confinement for quarantine and medical isolation, as well as facility-wide lockdowns where residents had to stay in their cell for days or weeks (Cassarino et al., 2023; Kramer et al., 2022; Song et al., 2023).

In 2020, our team conducted a survey of prisons and jails about COVID-19 changes to pregnancy care and services and found that quarantine practices were indeed more frequently applied to pregnant people and that at least one facility used solitary confinement for infection control—despite the fact that national guidelines recommend against solitary in pregnancy due to negative mental health impact and how it limits timely access to health care (Kramer et al., 2022; NCCHC, 2016). The practice of using solitary confinement as punishment is widespread throughout carceral facilities, and without properly differentiating between solitary confinement and medical isolation/quarantine *as a public health practice*, the latter can feel like punishment and undermine public health efforts (Cloud et al., 2020; Song et al., 2023). Yet, just as multiply marginalized, non-incarcerated communities faced more immediate concerns, even as COVID-19 exacerbated preexisting disparities, incarcerated pregnant people similarly described a “worsening” of already-intolerable conditions. The indiscriminate application of quarantine protocols to pregnant people reflects broader carceral logics that do not account for the wellbeing of pregnant and postpartum people and their infants, as evidenced by current practices of infant separation, a lack of support, and stressful living conditions.

Incarceration heightens postpartum vulnerability, since most incarcerated birthing people are separated from their newborns within 24–72 h after birth and suffer significant emotional turmoil as a result (Chambers, 2009). Similarly, our COVID-19 participants reported exacerbated suffering during the immediate postpartum period due to the isolation of quarantine and the inability to see their infants.

The emergence of the COVID-19 pandemic alongside the already punitive and degrading treatment of incarcerated pregnant and postpartum people suggests a syndemic effect, whereby the two crises each compounded the negative effects of the other (Caron & Adegboye, 2023; Fisher & Bubola, 2020; Tai et al., 2021). This mirrors how the pandemic deepened and further entrenched preexisting social, economic, and health disparities in community settings, as marginalized communities were disproportionately sickened and killed. News and other media characterized the pandemic as the greatest crisis of the present moment, but this did not reflect the reality of multiply marginalized communities who faced worsening and/or more immediately pressing concerns, including community or domestic violence, houselessness, food insecurity and hunger, an inability to access medical care, and/or deportation (Dahir, 2020; Dickerson, 2020; Eligon, 2021). Similarly, our incarcerated participants experienced the pandemic as yet one more crisis, compounding multiple other crises.

### Limitations

There are several limitations to this study. First, our recruitment was guided by the aims of the parent study established prior to the pandemic, which means we did not design the study to reach thematic saturation regarding participants’ experiences with the pandemic. More research specifically targeted towards the experience of pregnant and postpartum people during the COVID-19 pandemic is needed to understand the full impact of the pandemic on their experiences and care provision. These interviews only came from one U.S. prison, and so individuals at other facilities may have had different experiences. Additionally, all six participants interviewed during COVID-19 had only been at the prison for two months or less, and nine out of twelve were housed in Admissions (not General Population) at the time of the interview (see Table 2). If we had interviewed them after a longer period of incarceration or longitudinally, they may have had additional or different things to say about housing conditions as well as the impact of the pandemic. The same is also true if we had conducted in-depth interviews with individuals during the postpartum period, instead of during pregnancy. However, the rapid changes in public health recommendations, policies, and practices (especially as resources like testing kits and vaccines became more available) are extremely challenging to track via interview data, especially retrospectively. If possible, future research would triangulate interview reports with data on policies and practices from the facilities themselves. Although we asked about abortion, we were unable to assess the impact of the pandemic on pregnant people’s abilities to access it, as none of the participants described actively seeking one. However, research prior to the pandemic suggests that the coercive and autonomy-restricting dynamics inherent to incarceration may lead pregnant people to assume that they cannot access abortion regardless of their desires (Sufrin et al., 2023). The pandemic may have exacerbated this belief that abortion is inaccessible during incarceration. Finally, we were not able to assess how and to what degree the medical care for pregnant and postpartum people at this prison was impacted by the pandemic.

## Conclusions

The COVID-19 pandemic was characterized as a major crisis and primary threat to public health. Its rapid spread through U.S. carceral facilities illustrated how they are an amplified microcosm of broader health inequities, while also highlighting many inherent flaws of the carceral system in general (LeMasters et al., 2022). These data, along with newer information about COVID-19 prevention and treatment and about its course during pregnancy, suggest that carceral facilities and public health officials should specifically address the distinct pandemic-related needs of pregnant and postpartum people in custody, including greater transparency, more rational and effective quarantine policies, and avoiding solitary confinement in pregnancy. Additional qualitative and quantitative research is needed to assess not only the prevalence of pregnancy and related health issues in the U.S. carceral system, but also further documentation of the experiences and impacts of the COVID-19 pandemic on incarcerated pregnant and postpartum people. Public health and policy discussions planning for future pandemics and addressing the sequelae of mass incarceration must center the voices of directly impacted, pregnant individuals in order to be effective. Moreover, these data make the case for considering pregnant people as a priority population for diversion and early release in general, not just during public health emergencies.

## Data Availability

The data from this study is not publicly available, as it would compromise individual participants’ privacy and confidentiality.

## Footnotes

1 In this paper we use gendered terms and pronouns when referring to data on incarcerated women and to our participants, all of whom identified as women. However, we incorporate gender-inclusive terminology (e.g., pregnant people) throughout the paper as not all birthing people identify as women.

2 This prison did have a nursery program, but half our sample (n = 6) reported being ineligible, while others didn’t know if they’d been accepted into the program (*n* = 4). Two did not want to participate in the nursery program.

3 To try to prevent COVID transmissions, many hospitals around the country forbade support persons from accompanying birthing people during labor and birth, especially at the beginning of the pandemic.
